# Prevalence, multiple antibiotic resistance and virulence profile of methicillin-resistant *Staphylococcus aureus* (MRSA) in retail poultry meat from Edo, Nigeria

**DOI:** 10.3389/fcimb.2023.1122059

**Published:** 2023-03-02

**Authors:** Etinosa O. Igbinosa, Abeni Beshiru, Isoken H. Igbinosa, Abraham G. Ogofure, Temitope C. Ekundayo, Anthony I. Okoh

**Affiliations:** ^1^ Applied Microbial Processes and Environmental Health Research Group, Faculty of Life Sciences, University of Benin, Benin City, Nigeria; ^2^ Stellenosch Institute for Advanced Study (STIAS), Wallenberg Research Centre at Stellenbosch University, Stellenbosch, South Africa; ^3^ Department of Microbiology, College of Natural and Applied Sciences, Western Delta University, Oghara, Nigeria; ^4^ Department of Environmental Management and Toxicology, Faculty of Life Sciences, University of Benin, Benin City, Nigeria; ^5^ South African Medical Research Council (SAMRC) Microbial Water Quality Monitoring Centre, University of Fort Hare, Alice, Eastern Cape, South Africa; ^6^ Department of Microbiology, University of Medical Sciences, Ondo City, Ondo, Nigeria; ^7^ Department of Environmental Health Sciences, College of Health Sciences, University of Sharjah, Sharjah, United Arab Emirates

**Keywords:** resistant determinants, biofilm formation, cassette chromosomes, virulence, enterotoxin

## Abstract

**Introduction:**

*Staphylococcus aureus* causes staphylococcal food poisoning and several difficult-to-treat infections. The occurrence and dissemination of methicillin-resistance *S. aureus* (MRSA) in Nigeria is crucial and well documented in hospitals. However, findings on MRSA from meat in the country are yet to be adequately reported. The current study determined the prevalence, virulence profile and antibiogram characteristics of MRSA from a raw chicken product from retail outlets within Edo.

**Methods:**

A total of 368 poultry meat samples were assessed for MRSA using a standard culture-based approach and characterized further using a molecular method. The antimicrobial susceptibility profile of the isolates was determined using the disc diffusion method. The biofilm profile of the isolates was assayed via the crystal violet microtitre-plate method. Virulence and antimicrobial resistance genes were screened using polymerase chain reaction via specific primers.

**Results:**

Of the samples tested, 110 (29.9%) were positive for MRSA. All the isolates were positive for deoxyribonuclease (DNase), coagulase and beta-hemolysis production. Biofilm profile revealed 27 (24.55%) weak biofilm formers, 18 (16.36%) moderate biofilm formers, and 39 (35.45%) strong biofilm formers. The isolates harboured 2 and ≤17 virulence genes. Enterotoxin gene profiling revealed that 100 (90.9%) isolates harboured one or more genes. Resistance against the tested antibiotics followed the order: tetracycline 64(58.2%), ciprofloxacin 71(64.6%), trimethoprim 71(64.6%) and rifampin 103(93.6%). A total of 89 isolates were multidrug-resistant, while 3 isolates were resistant to all 22 antibiotics tested. The isolates harboured antimicrobial-resistant determinants such as methicillin-resistant gene (*mec*A), tetracycline resistance genes (*tet*K, *tet*L), erythromycin resistance genes (*erm*A, *erm*C), trimethoprim resistance gene (*dfr*K). All the staphylococcal cassette chromosome mec (*SCCmec*) IVa and *SCCmec* V positive isolates harboured the Panton-Valentine Leukocidin Gene (PVL).

**Conclusion:**

In conclusion, *S. aureus* was resistant to commonly used antibiotics; a concern to public health concerning the transmission of these pathogens after consuming these highlight the significance of antimicrobial and enterotoxigenic monitoring of *S. aureus* in food chains.

## 1 Introduction

The commensal association of *S. aureus* with food animals promotes antimicrobial resistance (Zehra et al., 2019). *Staphylococcus aureus* (*S. aureus*) has been reported to acquire resistance to antibiotics of choice, such as vancomycin (Tegegne et al., 2021). It has been listed as a ‘priority pathogens’ threatening public health by the World Health Organization (WHO) (WHO, 2017). Methicillin-resistance *S. aureus* (MRSA) is a significant aetiology of massive healthcare-associated MRSA (HA-MRSA) infections leading to antibiotic resistance crises worldwide (Abolghait et al., 2020). MRSA is of concern since it can cause difficult-to-treat fatal infections. Although there has been a decline in invasive HA-MRSA infections over recent years, community-associated MRSA (CA-MRSA) infections have heightened among the general populace. The molecular mechanism for developing oxacillin/methicillin resistance is inserting and acquiring staphylococcal chromosome cassette *mec* (*SCCmec*) determinants, which house antimicrobial resistance genes. Differences exist in the *SCCmec* types of *S aureus* strains, which may increase by independently acquiring the *mec* gene. Furthermore, HA-MRSA, livestock-associated MRSA (LA-MRSA), and CA-MRSA can contaminate human foods, causing cases of staphylococcal food poisoning (SFP) (Sergelidis and Angelidis, 2017). *S. aureus* food-borne disease outbreaks have been reported recently (Le et al., 2021).

Staphylococcal food poisoning (SFP) is associated with emetic activity, sepsis-related infections, pneumonia, and toxic shock syndrome (TSS) (Fisher et al., 2018). Upon contamination of food, food triggers enterotoxins production in *S. aureus*, which may persist in foodstuffs after heat decimation of the bacteria, thus causing SFP (Sergelidis and Angelidis, 2017). Staphylococcal enterotoxins (SEs) comprise a superfamily of ≥23 low-molecular-weight pyrogenic exotoxins that share functional and structural similarities. Staphylococcal enterotoxins can be classified into two categories depending on their ability to invoke emesis: newly confirmed enterotoxigenic-like proteins and classical SEs (A to E). Staphylococcal enterotoxins possess potent super antigenic activity and disrupt adaptive immunity by stimulating T cells, producing inflammatory cytokines (Fisher et al., 2018). The SE-encoding elements are mainly on different mobile genetic elements (MGEs), which can extensively result in prevalence variations of SEs among *S. aureus* isolates. In addition, SEs are controlled by various overlapping regulatory pathways that can be multiple by various environmental factors (Fisher et al., 2018).

The ingestion of 20–100 ng performed SEs in food caused SFP (Abolghait et al., 2020; Beshiru et al., 2022). The production of SEs essentially occurred in protein-rich foods, such as meat, after the growth of enterotoxigenic *S. aureus* strains at high cell densities under optimal temperatures and environmental conditions (Schelin et al., 2011; Igbinosa et al., 2020). Staphylococcal enterotoxins retain activity in *S. aureus*-contaminated food due to their high heat stability, protein denaturation tolerance, proteolytic enzyme activity resistance, freezing, drying, and low pH conditions (Abolghait et al., 2020). Additionally, they are acid-stable and retain their activity in the digestive tract (Fisher et al., 2018). Chicken products can be a potential reservoir of MRSA infections of zoonotic origin. Chicken meat not kept at refrigeration temperature can be contaminated with SEs-producing MRSA, which may create a health hazard. Consuming or handling contaminated food could result in the spread to humans (Igbinosa et al., 2021a; Beshiru et al., 2022). Sequel to concerns from the Nigerian government regarding food safety, significant proportions of data concerning the characteristics and Prevalence of MRSA, including SEs-producing MRSA in retail foods in Nigeria, is essential.

*S. aureus* is biofilm-producing bacteria and can perpetuate its contamination on contact surfaces in meat processing (Igbinosa et al., 2020; Abbasi et al., 2021; Beshiru et al., 2021) and could increase bacterial resistance and spread in farm animals, favor their persistence in the environment, and improve their survival in meat products. Some *S. aureus* lineages have adapted to chicken (Lowdera et al., 2009; Murray et al., 2017) and harbor multiple virulence and antimicrobial resistance (AMR) genes. Biofilm attachment involves bacterial surface component sensing adhesive and matrix molecules, whereas biofilm maturation consists of the expression of intercellular adhesion gene cluster (*ica*ABCD) operon-encoded polysaccharide adhesion molecules (Nemati et al., 2009; Bernier-Lachance et al., 2020). The importance of biofilm-forming MRSA in foods of animal origins is not adequately documented in Nigeria and other developing countries. Thus, the present study was designed to assay the occurrence and distribution of biofilm-associated determinants, virulence, and resistance elements in MRSA recovered from chicken carcasses in Nigeria.

The Prevalence of MRSA varies in different food, animals, and country of origin (Lim et al., 2010; Wu et al., 2019). In Nigeria, there is sparse information on the prevalence of MSRA in food and food animals, with few unrelated studies reporting MRSA infection rates (Beshiru et al., 2016; Igbinosa et al., 2016a; Igbinosa et al., 2016b; Igbinosa and Beshiru, 2019; Abolghait et al., 2020; Beshiru et al., 2021). *S. aureus* and MRSA have been recovered from poultry birds, frozen fish, humans, environmental samples, milk, pork, beef, ready-to-eat food, livestock, dressed chicken, pet, and stray dogs in Nigeria (Igbinosa et al., 2016a; Igbinosa et al., 2016b; Beshiru et al., 2021). However, reports on the molecular characterization of MRSA from chicken are scarce. A better understanding of the epidemiology and identifying the genetic profile of MRSA is crucial in developing preventive mechanisms against infectious of its origin. Likewise, understanding the genetics of MRSA circulating in different milieus is vital for its evolutional tracking in various niches. Hence, this study aimed to determine the antibiotic resistance, prevalence, enterotoxigenic, and other genetic profiles of MRSA in retail chicken meat sold at open markets in Nigeria.

## 2 Materials and methods

### 2.1 Sample collection

Frozen chicken carcass samples were obtained from cold rooms operating on a large scale from open markets in Edo, Nigeria. The selection of the markets was based on their strategic location in conjunction with consumer patronage. The cold rooms selected usually distribute chicken carcasses at a cheaper wholesale rate to retail traders who re-sell to direct consumers in their local markets. The sample size was determined *via* the sample size determination formula:


$$Sample\;\left(N\right)=\;\frac{{\left(Z_{1-\alpha /2}\right)}^{2}\;P\left(1-P\right)}{d^{2}}$$

N = Number of expected samples; Z_1-α/2_ = Standard normal variant at 5% type I error (P< 0.05); P = prevalence expected based on previous studies [1.3% (Bernier-Lachance et al., 2020), 9.89% (Baghbaderani et al., 2020), 13.9% (Abbasi et al., 2021), 20.5 (Li et al., 2019), 29.1% (Rortana et al., 2021), 35.4% (Tegegne et al., 2021), 66.67% (Savariraj et al., 2020), 89.5% (Lika et al., 2021)]; d = Complete precision or error (which is 5%). Thus, the expected size of the sample was ≥351. The markets sampled include Ikpoba Hill (*n*=30) [6.3496° N, 5.6609° E], New Benin (*n*=32) [6.3448° N, 5.6340° E], Oba (*n*=32) [6.3348° N, 5.6201° E], Santana (*n*=30) [6.2915° N, 5.6325° E], Aduwawa (*n*=32) [6.3688° N, 5.6849° E], Uselu (*n*=31) [6.3744° N, 5.6134° E], Ileha (*n*=30) [6.3460° N, 5.6097° E], Ekiosa (*n*=31) [6.3231° N, 5.6363° E], Oka (*n*=30) [6.2905° N, 5.6623° E], Egor (*n*=30) [6.3642° N, 5.6090° E], Oregbeni (*n*=30) [6.3501° N, 5.6592° E], and Ugbor (*n*=30) [6.2629° N, 5.6063° E] markets. The sampling was conducted from June 2018 to April 2019. Seasonal durations in Nigeria include a wet season characterized by heavy rainfall with a temperature reaching 35 ± 2°C (March to September) and a dry season characterized by low to no rainfall with a temperature reaching 39 ± 2°C (November to February). A total of 368 frozen fresh chicken meat samples were collected. In all cases, 50 g of samples were collected into a plastic tube from retail outlets within Benin City and transported on ice to the laboratory (Applied Microbial Processes & Environmental Health Research Laboratory, University of Benin, Nigeria).

### 2.2 MRSA isolation

Twenty-five grams of the sample was inoculated into 225 ml trypticase soy broth (Merck, Darmstadt, Germany), incubated at 37 °C for 24 h, and subcultured *via* streaking onto MRSA selective agar plate (CHROMagar™ MRSA-ITK Diagnostics BV, Netherlands) and incubated at 37°C for 24 h. Rose to mauve colonies on MRSA selective agar plates were presumptive MRSA isolates. The isolates were identified based on cultural, morphological, and biochemical tests such as Gram-reactions, 3% potassium hydroxide (3% KOH), catalase, coagulase, β-haemolysis, DNAse activity, anaerobic utilization of glucose and mannitol (Tallent et al., 2019). One colony per plate was purified in nutrient agar (Lab M, Lancashire, United Kingdom), further incubated for 18 h at 37°C, and preserved on nutrient agar slants at 4°C. The positive control used includes *S. aureus* (ATCC 12600).

### 2.3 Phenotypic confirmation of MRSA isolates

Phenotypic detection of MRSA was performed using cefoxitin disk assay (CLSI, 2020). For individual isolates, colonies of isolated *S. aureus* from an overnight-grown culture were transferred into the nutrient broth. *S. aureus* suspensions at a 0.5 McFarland standard equivalent density in nutrient broth were spread onto Mueller–Hinton agar (Lab M, Lancashire, UK) plate in duplicate with cefoxitin (30 µg), methicillin (5 µg), cloxacillin (5 µg), and oxacillin (1 µg) disc (Mast Diagnostics, UK). The plates were incubated at 37°C for 24 h. Isolates identified with cefoxitin resistance (≤ 21 mm zone diameter) were categorized as MRSA.

### 2.4 PCR detection and characterization of the MRSA isolates

The genomic DNA of the MRSA isolates and positive *S. aureus* (*S. aureus* ATCC 12600) control were extracted using the DNA MiniPrep kit following the manufacturer’s instructions. The polymerase chain reaction (PCR) uses to identify *Staphylococcus aureus* using specific primers (**Supplementary Table 1**) and the PCR reaction previously described (Brakstad et al., 1992) by targeting the *nuc* gene. AMR genes such as methicillin resistance (*mecA*), trimethoprim (*dfrG*, *dfrK*, *dfrD*), aminoglycosides [*ant(4´)-Ia*, *aac(6´)-Ie-aph(2´´)-Ia*, *aph(3´)-IIIa*], chloramphenicol (*cat::pC194*, *cat::pC223*, *cat::pC221*), erythromycins (*ermA*, *ermB*, *ermC*), tetracyclines (*tetO*, *tetM*, *tetL*, *tetK*) and beta-lactamase (*BlaZ*); and virulence genes such as intercellular adhesion protein (*icaA*, *icaB*, *icaC*, *icaD*), toxic shock syndrome toxin 1 (*tsst-1*), exfoliative toxin (*eta*, *etb*), enterotoxins (*sea* to *seu*), haemolysins (*hla*, *hlb*), Panton-Valentine Leucocidin (PVL), staphylococci protein A (*spa*), and coagulase (*coa*) were amplified as described previously (Jackson and Landolo, 1986; Dale et al., 1995; Frenay et al., 1996; Monday and Bohach, 1999; Martineau et al., 2000; Fueyo et al., 2005). SCC*mec* I to V and subtype SCC*mec* (IVa to d) of the MRSA isolates was carried out using PCR as described previously (Okuma et al., 2002; Ma et al., 2005; Zhang et al., 2005) using specific primer sets in **Supplementary Table 1**. PCR products were performed in a 1% agarose gel electrophoresis for 45 min at 110 V, viewed after staining with ethidium bromide in a transilluminator (Vilber Lourmat, EBOX VX5, France).

### 2.5 Antimicrobial susceptibility testing

The antibiogram profiling of the MRSA isolates was conducted using the Kirby-Bauer disc diffusion procedure. Antibiotics used includes Penicillin G (10 units), ceftaroline (30µg), gentamicin (10µg), amikacin (30µg), kanamycin (30µg), azithromycin (15µg), clarithromycin (15µg), erythromycin (15µg), doxycycline (30µg), minocycline (30µg), tetracycline (30µg), ciprofloxacin (5µg), levofloxacin (5µg), moxifloxacin (5µg), nitrofurantoin (300µg), clindamycin (2µg), trimethoprim-sulfamethoxazole (1.25/23.75µg), trimethoprim (5µg), chloramphenicol (30µg), sulfonamides (300µg), linezolid (30µg) and rifampin (5µg). The interpretation of the zone of inhibitions of the isolates as resistance, intermediate, or susceptibility was based on the Clinical and Laboratory Standard Institute’s interpretative chart (CLSI, 2020) to determine the sensitivity, intermediate and resistance profiles of the isolates to the antibiotics used. For vancomycin (1-32 µg/mL), oritavancin (0.12-0.50 µg/mL), teicoplanin (4-64 µg/mL), daptomycin (0.5-4 µg/mL), and tedizolid (0.25-4 µg/mL) antibiotics, the minimum inhibitory concentration (MIC) procedure was adopted, and data were interpreted based on interpretive categories and MIC breakpoints, µg/mL (CLSI, 2020). Multidrug resistance and multiple antibiotic resistance index were determined as described elsewhere (Igbinosa et al., 2022).

### 2.6 Biofilm formation profile of the MRSA isolates

Biofilm formation assay was carried out by suspending pure MRSA colonies in 4.5 mL tryptone soy broth (TSB) and incubating for 18 h at 37°C. After that, the cells were harvested at 12,000 rpm for 2 min., washed, re-suspended in phosphate-buffered solution at pH 7.2), and adjusted to 0.5 McFarland standards. A 20 mL of the suspended cell inoculant and 80 mL TSB were introduced into sterile 96-welled polystyrene microtitre plates to assess Staphylococci adherence onto a solid matrix/medium well-contained as described previously (Igbinosa et al., 2022). The negative and positive control was a well-containing TSB broth and *S. aureus* ATCC 12600, respectively. Each assay was done in independent biological triplicate. Biofilm-producing ability of the isolates was defined as non-producing/negative (ODi< ODc), weak/poor-producing (ODc< ODi<0.1), moderate/intermediate-producing (ODi ¼ 0.1< 0.12), or strong producer (ODi>0.12) as described somewhere else (Igbinosa et al., 2022).

## 3 Results

### 3.1 MRSA prevalence from chicken meat

From the 368 samples screened, 110(29.9%) were positive for MRSA *via* resistance (≤ 21 mm zone diameter) to the cefoxitin disc test. As such, representative isolates from the 110 samples were carefully screened using the *S. aureus* specific primer (*nuc* gene), where a total of 110 isolates were detected (**Table 1**).

**Table 1:** Phenotypic and genotypic virulence profile of the MRSA isolates.

Coded MRSA	Phenotypic virulence				Virulence genes													Enterotoxin genes																	
	DNase	Coagulase	β-hemolysis	Biofilm	*Nuc*	*coa*	*spa*	PVL	*hla*	*hlb*	*tsst*-1	*etb*	*eta*	*ica*D	*ica*C	*ica*B	*ica*A	*sea*	*seb*	*sec*	*sed*	*see*	*seg*	*seh*	*sei*	*sej*	*sek*	*sel*	*sem*	*sen*	*seo*	*sep*	*seq*	*ser*	*seu*
SA001				+																															
SA002				+																															
SA003				+++																															
SA004				+																															
SA005				+++																															
SA006				–																															
SA007				+																															
SA008				+++																															
SA009				+++																															
SA010				+++																															
SA011				–																															
SA012				–																															
SA013				+																															
SA014				++																															
SA015				+++																															
SA016				+++																															
SA017				++																															
SA018				–																															
SA019				+++																															
SA020				+++																															
SA021				+++																															
SA022				++																															
SA023				+++																															
SA024				–																															
SA025				+																															
SA026				++																															
SA027				++																															
SA028				+++																															
SA029				+++																															
SA030				–																															
SA031				–																															
SA032				++																															
SA033				++																															
SA034				+++																															
SA035				++																															
SA036				+++																															
SA037				++																															
SA038				+++																															
SA039				–																															
SA040				+++																															
SA041				–																															
SA042				+																															
SA043				+++																															
SA044				+																															
SA045				+++																															
SA046				–																															
SA047				–																															
SA048				++																															
SA049				+																															
SA050				+																															
SA051				+++																															
SA052				+++																															
SA053				+																															
SA054				+																															
SA055				–																															
SA056				–																															
SA057				++																															
SA058				–																															
SA059				+																															
SA060				+++																															
SA061				++																															
SA062				–																															
SA063				+++																															
SA064				+++																															
SA065				–																															
SA066				+																															
SA067				+																															
SA068				++																															
SA069				++																															
SA070				+++																															
SA071				–																															
SA072				–																															
SA073				–																															
SA074				+																															
SA075				–																															
SA076				+++																															
SA077				–																															
SA078				+																															
SA079				+																															
SA080				+++																															
SA081				+																															
SA082				+++																															
SA083				+++																															
SA084				+																															
SA085				+																															
SA086				–																															
SA087				+++																															
SA088				+																															
SA089				+++																															
SA090				+++																															
SA091				+																															
SA092				–																															
SA093				+																															
SA094				+++																															
SA095				+																															
SA096				+																															
SA097				–																															
SA098				+++																															
SA099				++																															
SA100				+++																															
SA101				+++																															
SA102				++																															
SA103				–																															
SA104				+++																															
SA105				++																															
SA106				+++																															
SA107				+++																															
SA108				++																															
SA109				+																															
SA110				–																															

Black box colour = positive; white box colour = negative; +++ = strong biofilm formation; + = weak biofilm formation; - = no biofilm formation; ++ = moderate biofilm formation.

### 3.2 Phenotypic virulence factors of the MRSA isolates

All MRSA isolates from this study were 100% positive for DNase, coagulase, and beta-hemolysis production (**Table 1**). The biofilm profile of the isolates revealed that 27(24.55%) were weak biofilm producers, 18(16.36%) were moderate biofilm producers, 39(35.45%) were strong biofilm producers, while 26(23.64%) were negative for biofilm formation (**Table 2**). An overall total of 84(76.36%) of the isolates were biofilm formers (**Table 1**).

### 3.3 Occurrence of virulence determinants from the MRSA isolates

The occurrence of virulence genes screened in this study is as follows: *coa* 110(100%), *spa* 98(89.1%), *hla* 110(100%), *pvl* 50(45.5%), *hlb* 23(20.95), *sea* 27(24.6%), *seb* 16(14.6%), *sec* 19(17.3%), *sed* 14(12.7%), *see* 13(11.8%), *seg* 39(35.5%), *seh* 13(11.8%), *sei* 11(10%), *sej* 32(29.1%), *sek* 4(3.6%), *sel* 4(3.6%), *sep* 20(18.2%), *ser* 13(11.8%), *tsst* 16(14.6%), *etb* 17(15.5%), *eta* 17(15.5%), *ica*A 45(40.9%), *ica*B 44(40%) (**Table 1**). The *sem*, *sen*, *seo*, *seq*, *seu*, *ica*D and *ica*C were not detected. Overall, staphylococci isolates harbored a minimum of 2 virulence genes and a maximum of 17 virulence genes (**Table 1**). All the isolates that possessed the adherence determinant (*icaA* and *icaB*) formed biofilms phenotypically. All the isolates that possessed the *hla* and *hlb* genes were β-hemolytic phenotypically on blood agar. All the isolates in the study were coagulase positive *via* biochemical process and possessed the *coa* determinant. Enterotoxin gene (18 *sea*-*seu* genes) profiling of the isolates revealed that 90.9% (100/110) of the isolates were enterotoxigenic, harboring either one or more genes of the 13 genes that were positive. The chain of enterotoxin gene occurrence was *seg>sej>sea>sep>sec>seb>sed> see,she,ser>sei>sek,sel.* All the *ica* gene-carrying isolates were biofilm formers (**Table 1**). The combined occurrence of virulence genes include: *hla+hlb* 23(20.9%), *eta+etb* 17(15.5%), *icaA+IcaB* 37(33.6%), *pvl+hla+hlb* 23(20.9%), *pvl+tsst-1* 16(14.5%) (**Table 1**).

### 3.4 Antimicrobial susceptibility profile and determinants of the MRSA isolates

The resistance profile of the *S. aureus* in **Table 2** to the antibiotics tested is as follows: penicillin G 110(100%), clarithromycin 53(48.2%), doxycycline 58(52.7%), minocycline 53(48.2%), tetracycline 64(58.2%), ciprofloxacin 71(64.6%), levofloxacin 84(76.4%), moxifloxacin 88(80%), clindamycin 62(56.4%), sulfonamides 53(48.2%), trimethoprim 71(64.6%), and rifampin 103(93.6%). The sensitivity profile of the isolates in **Table 3** is as follows: ceftaroline 54(49.1%), gentamicin 51(46.4%), amikacin 64(58.2%), kanamycin 56(50.9%), nitrofurantoin 100(90.9%), trimethoprim-sulfamethoxazole 58(52.7%), chloramphenicol 78(70.9%), and linezolid 83(75.5%). All the isolates were vancomycin, oritavancin, teicoplanin, daptomycin, and tedizolid-sensitive (**Table 2**).

**Table 2:** Antimicrobial resistance phenotype of the MRSA isolates.

Coded MRSA	MAR Index	Antibiotics																										
		PEN	VAN	CPT	DAP	GEN	AMI	KAN	AZM	CLR	ERY	ORI	TEI	DOX	MIN	TET	CIP	LVX	MXF	NIT	CLI	SXT	SSS	TMP	CHL	LNZ	TED	RIF
SA001	0.11																											
SA002	0.29																											
SA003	0.74																											
SA004	0.26																											
SA005	0.63																											
SA006	0.04																											
SA007	0.3																											
SA008	0.7																											
SA009	0.67																											
SA010	0.52																											
SA011	0.07																											
SA012	0.04																											
SA013	0.22																											
SA014	0.48																											
SA015	0.74																											
SA016	0.67																											
SA017	0.41																											
SA018	0.07																											
SA019	0.74																											
SA020	0.56																											
SA021	0.74																											
SA022	0.44																											
SA023	0.74																											
SA024	0.07																											
SA025	0.15																											
SA026	0.44																											
SA027	0.7																											
SA028	0.56																											
SA029	0.7																											
SA030	0.04																											
SA031	0.07																											
SA032	0.48																											
SA033	0.81																											
SA034	0.7																											
SA035	0.48																											
SA036	0.7																											
SA037	0.41																											
SA038	0.59																											
SA039	0.04																											
SA040	0.78																											
SA041	0.07																											
SA042	0.26																											
SA043	0.67																											
SA044	0.15																											
SA045	0.78																											
SA046	0.11																											
SA047	0.04																											
SA048	0.41																											
SA049	0.26																											
SA050	0.19																											
SA051	0.7																											
SA052	0.74																											
SA053	0.15																											
SA054	0.22																											
SA055	0.07																											
SA056	0.11																											
SA057	0.56																											
SA058	0.11																											
SA059	0.22																											
SA060	0.78																											
SA061	0.56																											
SA062	0.07																											
SA063	0.48																											
SA064	0.74																											
SA065	0.07																											
SA066	0.19																											
SA067	0.19																											
SA068	0.59																											
SA069	0.56																											
SA070	0.74																											
SA071	0.19																											
SA072	0.07																											
SA073	0.11																											
SA074	0.29																											
SA075	0.04																											
SA076	0.81																											
SA077	0.04																											
SA078	0.37																											
SA079	0.19																											
SA080	0.7																											
SA081	0.19																											
SA082	0.67																											
SA083	0.78																											
SA084	0.15																											
SA085	0.26																											
SA086	0.07																											
SA087	0.67																											
SA088	0.19																											
SA089	0.78																											
SA090	0.7																											
SA091	0.22																											
SA092	0.07																											
SA093	0.22																											
SA094	0.74																											
SA095	0.37																											
SA096	0.29																											
SA097	0.07																											
SA098	0.78																											
SA099	0.29																											
SA100	0.81																											
SA101	0.59																											
SA102	0.37																											
SA103	0.07																											
SA104	0.78																											
SA105	0.29																											
SA106	0.59																											
SA107	0.74																											
SA108	0.26																											
SA109	0.29																											
SA110	0.07																											

**PEN: ERY**: Erythromycin 15µg; Penicillin G 10 units; **CPT**, Ceftaroline 30µg; **GEN**, Gentamicin 10µg; **AMI**, Amikacin 30µg; **KAN**, Kanamycin 30µg; **AZM**, Azithromycin 15µg; **CLR**, Clarithromycin 15µg; **DOX**, Doxycycline 30µg; **MIN**, Minocycline 30µg; **CIP**, Ciprofloxacin 5µg; **LVX**, Levofloxacin 5µg; **MXF**, Moxifloxacin 5µg; **NIT**, Nitrofurantoin 300µg; **CLI**, Clindamycin 2µg; **DAP**, Daptomycin; **SXT**, Trimethoprim-sulfamethoxazole 1.25/23.75µg; **SSS**, Sulfonamides 300µg; **TMP**, Trimethoprim 5µg; **CHL**, Chloramphenicol 30µg; **RIF**, Rifampin 5µg; **VAN**, Vancomycin; **ORI**, Oritavancin; **TET**, Tetracycline 30µg; **TEI**, Teicoplanin; **LNZ**, Linezolid 30µg; and **TED**, Tedizolid; Red colour = resistance; green colour = sensitive; yellow colour = intermediate.

**Table 3:** Antimicrobial resistance and *SCCmec* determinants of the MRSA isolates.

Coded MRSA	Antimicrobial resistance determinants																*SCCmec*								
	*mec*A	*bla*Z	*tet*K	*tetL*	*erm*A	*erm*B	*erm*C	*aac(6´)-Ie-aph(2´´)-Ia*	*ant(4´)-Ia*	*aph(3´)-IIIa*	*cat::p*C194	*cat::p*C221	*cat::p*C223	*dfr*D	*dfrK*	*dfr*G	Type I	Type II	Type III	Type IVa	Type IVb	Type IVc	Type IVd	Type IVh	Type V
SA001																									
SA002																									
SA003																									
SA004																									
SA005																									
SA006																									
SA007																									
SA008																									
SA009																									
SA010																									
SA011																									
SA012																									
SA013																									
SA014																									
SA015																									
SA016																									
SA017																									
SA018																									
SA019																									
SA020																									
SA021																									
SA022																									
SA023																									
SA024																									
SA025																									
SA026																									
SA027																									
SA028																									
SA029																									
SA030																									
SA031																									
SA032																									
SA033																									
SA034																									
SA035																									
SA036																									
SA037																									
SA038																									
SA039																									
SA040																									
SA041																									
SA042																									
SA043																									
SA044																									
SA045																									
SA046																									
SA047																									
SA048																									
SA049																									
SA050																									
SA051																									
SA052																									
SA053																									
SA054																									
SA055																									
SA056																									
SA057																									
SA058																									
SA059																									
SA060																									
SA061																									
SA062																									
SA063																									
SA064																									
SA065																									
SA066																									
SA067																									
SA068																									
SA069																									
SA070																									
SA071																									
SA072																									
SA073																									
SA074																									
SA075																									
SA076																									
SA077																									
SA078																									
SA079																									
SA080																									
SA081																									
SA082																									
SA083																									
SA084																									
SA085																									
SA086																									
SA087																									
SA088																									
SA089																									
SA090																									
SA091																									
SA092																									
SA093																									
SA094																									
SA095																									
SA096																									
SA097																									
SA098																									
SA099																									
SA100																									
SA101																									
SA102																									
SA103																									
SA104																									
SA105																									
SA106																									
SA107																									
SA108																									
SA109																									
SA110																									

Black box colour = positive; white box colour = negative.

A total of 89(80.9%) isolates were multidrug resistant been resistant to ≥1 antibiotic in ≥3 antimicrobial classes. A total of 3 isolates were resistant to 22/27(81.5%) antibiotics used in this study. All isolates were resistant to ≥ 1 antibiotic, while 89(80.9%) isolates were resistant to ≥ 3 antibiotics (**Table 2**). Of the isolates, 103(93.6%) were resistant to ≥2 antibiotics. MAR index of the isolates ranged from 0.04 – 0.81. Isolates with MAR index >2 were 73(66.4%). The highest resistant phenotype was PEN^R^+CPT^R^+GEN^R^+AMI^R^+KAN^R^+AZM^R^+CLR^R^+ERY^R^+DOX^R^+MIN^R^+ TET^R^+CIP^R^+LVX^R^+MXF^R^+NIT^R^+CLI^R^+SXT^R^+SSS^R^+TMP^R^+CHL^R^+LNZ^R^+RIF^R^ with a MAR index of 0.81. A total of 57 resistance phenotypic patterns were observed amongst all the isolates studied (**Table 2**).

The antibiotic-resistant genes detected includes: *mec*A 91/110(82.7%), *bla*Z 91/110(82.7%), *tet*K 64/110(58.2%), *tet*L 23/110(20.9%), *erm*A 55/110(50%), *erm*B 9/110(8.2%), *erm*C 21/110(19.1%), *aac(6´)-Ie-aph(2´´)-Ia* 37/110(33.6%), *ant(4´)-Ia* 28/110(25.5%), *aph(3´)-IIIa* 26/110(23.6%), *cat::p*C194 20/110(18.2%), *cat::p*C221 12/110(10.9%), *cat::p*C223 12/110(10.9%), *dfr*D 64/110(58.2%), *dfr*K 22/110(20%), *dfr*G 38/110(34.5%) (**Table 3**). Other resistant genes, such as *tet*M and *tet*O, were not detected (**Table 3**). A total of 91(82.7%) isolates harbored ≥1 antimicrobial resistance gene, while 19(17.3%) of the isolates didn’t harbor any antimicrobial resistance gene (**Table 3**). The combined occurrence of antimicrobial resistance genes includes: *mecA+blaZ* 91(82.7%), *tetK+tetL* 23(20.9%), *ermA+ermB* 9(8.2%), *ermA+ermB+ermC* 3(2.7%), *aac(6´)+ ant(4´)+aph(3´)* 25(22.7%), *aac(6´)+ant(4´)* 30(27.3%), *cat::pC194+cat::pC221+ cat::pC223* 7(6.4%), *cat::pC194+ cat::pC221* 12(10.9%), *dfrD+dfrK+dfrG* 17(15.5%), *dfrD+dfrK* 22(20%), *dfrD+dfrG* 37(33.6%) (**Table 3**).

The distribution of *SCCmec* is as follows: *SCCmec* III 18(16.4%), *SCCmec* IVa 33(30%) and *SCCmec* V 17(15.5%) (**Table 3**). Other *SCCmec* screened (*SCCmec* I, II, IVb, IVc, IVd, and IVh) were not detected (**Table 3**). The combined occurrence of *SCCmec* and *pvl* genes includes *SCCmec* IVa+*pvl* 33(30%) and *SCCmec* V+*pvl* 17(15.5%). All the *SCCmec* IVa and *SCCmec* V isolates also harbored the PVL gene, while all the *SCCmec* III isolates didn’t harbor the PVL gene (**Table 3**).

## 4 Discussion

Methicillin-resistance *S. aureus* from poultry meat poses a public health risk that can transmit to humans *via* the handling or consumption of contaminated poultry meat. Lower prevalence rates than those of our study (ranging from 0 – 27%) have been reported previously in North and South America, Europe, and Asia (Tang et al., 2017; Bernier-Lachance et al., 2020). Higher prevalence rates (35.4%) compared to our findings have also been documented in the Czech Republic (Tegegne et al., 2021). Differences in geographical locations, handling practices, sample size variations, seasonal variations, management practices, and methods of the experiment have been reported to cause differences in the prevalence of MRSA (Abbasi et al., 2021). These results highlight the necessity to mitigate the risk of MRSA transmission dynamics *via* meat products to humans.

Bernier-Lachance et al. (2020) isolates formed biofilm *via* the microtiter plate assay, which was higher than our study’s biofilm formers (76.36%). Biofilm occurrence meant that the MRSA isolates have adhesive potentials to the host’s extracellular matrix; these likely favor zoonotic potential, persistence, and colonization. Nigeria, where a significant proportion of the population is non-vegetarian, suggests that a large population is t risk of meat-borne hazards. Szczuka et al. (2013) reported that 76% of the biofilm-forming strains had the *icaA* gene (Yazdani et al., 2006), which was lower than the 100% *icaA* gene-carrying isolates that formed biofilm in our study. High distribution of biofilm formation genes (*icaA, icaB, icaC, icaD*) have been demonstrated previously (Nemati et al., 2009; Abbasi et al., 2021), which was different from our study where *icaC* and *icaD* were not detected. The *icaABCD* genes encode intercellular polysaccharide adhesion, which shields *S. aureus* in difficult environmental circumstances such as immune responses, antimicrobials, and antiseptic agents. Higher detection rates of *icaABCD* genes have been reported (Abbasi et al., 2021). Biofilm production ability, presence of virulence determinants, and antimicrobial capacity in the genome of MRSA constitute a severe risk to public health.

In addition to infection/colonization, MRSA strains have caused SFP outbreaks (Sergelidis and Angelidis, 2017). Most MRSA isolates by Wu et al. (2019) were MDR and harbored ≥1 SE gene, similar to our findings. Lower profiling of SEs (13 - 83%) has been documented (Kitai et al., 2005; Li et al., 2018; Savariraj et al., 2020; Tegegne et al., 2021) compared to those of our study. The chain of distribution of the enterotoxin genes from our study was *seg>sej>sea>sep>sec>seb>sed>see,she,ser> sei>sek,sel.* This was different from those from other studies, such as *seb>seg>sei>sec>sed>sej* (Savariraj et al., 2020). Kitai et al. (2005) reported the distribution of *seb>sea>sec>sed*. Previous studies have found that *sea* (Nemati, 2014), *seg*, and *sei* (Pu et al., 2011) were the prevalent toxin gene somewhat similar to our study, where *seg* was the most predominant, followed by *sej, sea*, and others. In the present study, non-classical enterotoxin genes *seg* and *sej* had higher prevalence with the exemption of classical enterotoxin *sea*. Previously, newly described enterotoxin genes *seg*, *sem*, *sei*, and *sen* were found among *S. aureus* isolates with the exemption of classical enterotoxin genes known as major etiological factors in SFP (Hwang et al., 2007). Mosaic structure in the pathogenicity islands can contribute to the shuffling and rearrangement of the enterotoxin genes in *S. aureus* (Banaszkiewicz et al., 2019).

Savariraj et al. (2020) reported that none of their *S. aureus* isolates harbored *see sea*, and *seh* either alone or in combination, which negates the findings from our study as *sea* and *see* were detected independently or in variety. Higher prevalence of *seb* gene in MRSA and *S. aureus* in retail chicken carcasses have been reported (Kitai et al., 2005; Abolghait et al., 2020). Among the SEs, *seb* and *sea* are the best elucidated. Among well-known bacterial superantigens connected with SFP, asthma, atopic dermatitis, nasal polyps, and toxic shock syndrome in humans, SEB is the most potent (Fries and Varshney, 2013). Abolghait et al. (2020) reported that the isolate positive for the *sed* gene did not harbor the *tsst* gene, which negates the finding from our study where some of the isolates carried the *sed+tsst* gene. No combinations of >1 of the tested SE genes (*sea, sec*, *seb*, and *sed*) were found by Abolghait et al. (2020), which was also different from our findings where there were multiple combinations of SEs genes that could be attributed to the larger SEs gene pool we screened (*sea* - *seu*) which aligns with other studies (Titouche et al., 2020). Most of the enterotoxigenic isolates by Abolghait et al. (2020) encoded the *etb* and *tsst-1* genes which were similar to our findings. Wu et al. (2019) reported lower *tsst-1* gene detection (3.70%) compared to ours (14.6%). None of the MRSA isolates from previous studies (Bernier-Lachance et al., 2020) harbor genes encoding *eta*, *etb*, and *tsst-1*, which negates our findings as these genes were detected in some of the isolates. The SEs genotypes *sea*–*seg*–*sei* or *seg*– *sei*, which also exists from our study, are known to be associated with SFP outbreaks (Kérouanton et al., 2007). The existence of *hlb* and *hla* genes in MRSA isolates is crucial for SFP. Ariyanti et al. (2011) showed that the *hla* and *hlb* genes were ubiquitous among *S. aureus* isolated from food animals, similar to our study’s findings.

All MRSA isolates in our study harbored virulence determinants pivotal in toxin production, invasion, adhesion, immune modulation, tissue destruction, leucocyte, and erythrocytes lysis. Enterotoxin genes are borne on MGEs, plasmids (*seb*), bacteriophages (*sea*), or pathogenicity islands (*sec*) (Argudin et al., 2010), which explains their absence or presence in individual isolates either by vertical or acquisition transmission of genes respectively. The virulence genes detected in S. Aureus and MRSA in the current study have been documented in CA-MRSA isolates in humans in hospital settings (Pokhrel et al., 2016). The occurrence of these potential pathogenic MRSA isolates in chicken meat portends their role as a threat to public health. The Prevalence of SEs gene in *S. aureus*/MRSA varies from country to country and might likely reflect geographical differences and ecological differences in strains’ origins (Li et al., 2018; Abolghait et al., 2020).

Previous studies have reported lower MDR MRSA isolates within the range of 39.17 - 70.2% (Li et al., 2019; Zehra et al., 2019) compared to 80.9% from our study; while the MAR index was reported as 0.23% (Amoako et al., 2019) which is lower compared to most of the isolates from our study. It was reported previously that 97.1% of isolates were resistant to ≥1 antimicrobial agent (Li et al., 2019), which negates our findings as all isolates were resistant to ≥1 antimicrobial. A result from Iran revealed that 96.0% of isolates were resistant to ≥2 antimicrobials (Nemati, 2013), similar to the 93.6% reported in our study. Resistance to penicillin, ciprofloxacin, tetracycline, erythromycin and kanamycin has commonly been reported from meat samples (Li et al., 2019; Zehra et al., 2019). None of the *S. aureus* strains from previous studies (Tang et al., 2017; Li et al., 2019) was found to be resistant to nitrofurantoin, and less than ten strains were resistant to rifampicin, trimethoprim, chloramphenicol, teicoplanin or gentamicin. This was different from the findings from our study, where the isolates were resistant to the antibiotics mentioned with the exemption of teicoplanin, where no resistance was observed. However, from previous studies, low resistance rates varying from 2–9% were observed for clindamycin, oxacillin chloramphenicol, and ceftriaxone (Zehra et al., 2019), which negates the findings from our study. No vancomycin-resistant isolate was found in this study, similar to Okorie-Kanu et al. (2020).

Approximately 38 unique resistance phenotypic patterns were found among the chicken isolates by Zehra et al. (2019), which was lower than the 57-resistance phenotypic pattern found in our study. Antibiotics such as tetracyclines, fluoroquinolones, macrolides, and sulfonamides are important for human health and are listed by the WHO as critically essential antimicrobials (WHO, 2017). The marked resistance to such antimicrobials is perhaps not surprising since these drugs are inexpensive, orally administered, and are available from diverse sources where they are sold with or without prescription in Nigeria (Okorie-Kanu et al., 2020). The indiscriminate use of these antibiotics in food animal production is a cause for concern culminating in the upsurge of MDR *S. aureus* (Beshiru et al., 2016; Imanah et al., 2017; El-Ashker et al., 2020; Beshiru et al., 2021). The low levels of biosecurity practices compliance and poor husbandry practices promote indiscriminate use and overdependence of these antibiotics in water and feed as growth promoters and for prophylaxis purposes in poultry farms in Nigeria (Oviasogie et al., 2016; Igbinosa et al., 2021b; Igbinosa et al., 2023).

Similar reports of resistance to fluoroquinolones have been documented from retail meat products in South Africa, Ghana, and Bangladesh (Mkize et al., 2017). Fortunately, MRSA isolates in our study were susceptible to linezolid, vancomycin, daptomycin, tedizolid, teicoplanin, oritavancin, and nitrofurantoin in line with a previous report (Okorie-Kanu et al., 2020). These are the priority and critically essential antibiotics in human medicine (WHO, 2019). The high sensitivity observed could be because these drugs lack veterinary preparations and aren’t routinely used in a clinical setting. The efficient regulation or termination of antibiotic usage in food animals has decreased resistance to zoonotic bacteria in developed countries (Levy, 2014; El-Ashker et al., 2020). There is a need for the urgent execution of appropriate food safety strategies across all decision-makers, policy-makers, and stakeholders in environmental, animal, and human health to address the public health menace of antimicrobial resistance.

Mobile genetic elements (MGEs) carry virulence traits that encode *S. aureus* accessory genes (*tsst-1*, *eta* and *etb)* (Xia and Wolz, 2014). Therefore, upon acquiring MGEs conferring virulent traits, commensal *S. aureus* may become a virulent/pathogenic strain pathogen under favorable conditions (Sergelidis and Angelidis, 2017). A total of 82.7% of the cefoxitin-resistant MRSA had the *mecA* gene from our study. Lower *mecA* gene occurrence (5.5%) of examined samples has been reported previously with phenotypic MRSA-positive isolates in Egypt (Abolghait et al., 2020). Higher *mecA* gene occurrence (100%) from cefoxitin-resistant MRSA-positive isolates has been documented in Poland, Oklahoma, and China (Li et al., 2019). Some isolates were cefoxitin-sensitive and lacked *mecA* or *mecC* genes but were oxacillin-resistant (Shore and Coleman, 2013). There has also been a report of *S. aureus* isolates (5.07%) phenotypically resistant to oxacillin from India but genotypically lacking *mecA* gene (Zehra et al., 2019). Such a pattern may be attributed to β-lactamases’ hyperproduction of and elicitation of the variant of *mecA* gene and penicillin-binding protein with an altered binding affinity (Laurent et al., 2012).

A significant proportion of the β-lactamase-encoding gene (*blaZ)* was found among MRSA isolates in the current study in addition to *mecA*. Phenotypic resistance to gentamicin, tetracycline, and erythromycin was supported by detecting *tet*K, *aphA3* and *ermA/ermB/ermC*. Zehra et al. (2019) isolated carried resistance genes (*blaZ*, *mecA*, *aacA*-*aphD*, *ermB*, *ermC*, *tetK*, *tetL*, and *tetM*) similar to the findings of our study. All the tested MRSA by Bernier-Lachance et al. (2020) harbored a much higher *dfrG* gene (which confers resistance to trimethoprim) compared to our research. Abbasi et al. (2021) reported that ≥1 isolate carries one of the following resistance genes *blaZ*, *mecA*, *tetK*, *linA*, *tetM*, *ermA*, *ermB*, and *aacA*-*D* which was much higher than the 82.7% of the isolates from our study. The presence of the *tetK* and *tetM* genes similar to our finding explained the tetracycline resistance phenotype observed by Bernier-Lachance et al. (2020).

The *SCCmec* typing from our study showed that few belonged to the human-associated (HA) clones’ type *SCCmec* III, with the majority belonging to the CA-MRSA SCCmec IVa, similar to a previous study (Li et al., 2019). All MRSA with *SCCmecV* also harbored the *pvl* gene identical to the *SCCmecV*+*pvl* variants by Zehra et al. (2019), which are molecular markers for CA-MRSA, indicating their presence in food of animal origin. Our study’s panton-valentine leukocidin (PVL) detection was slightly higher than previously detected by Okorie-Kanu et al. (2020). Kim et al. (2015) reported MRSA-*SCCmecV*+*pvl* as the commonest MRSA isolate from meat samples (Kim et al., 2015). *SCCmec* elements of types IV and V are the commonly found *SCCmec* types in CA-MRSA (Abdulgader et al., 2015). *SCCmec* types IVa and V have been documented in HA-MRSA in Nigeria (Ghebremedhin et al., 2009).

## 5 Conclusion

The MRSA recovered demonstrated MDR potential while harboring potent enterotoxin determinants and other virulence traits that could be detrimental to human health. The MRSA isolates also showed biofilm-forming capacity, which could make them more prone to antimicrobial resistance and persist on biotic and abiotic surfaces. The findings highlight the significance of surveillance studies and the need to continuously monitor the food chain for foods of animal origin for the occurrence and spread of MRSA superbugs. Our findings have revealed that raw chicken meat from Nigeria is the reservoir of MRSA. This study has also raised concerns about MRSA transmission after consuming contaminated chicken products. These findings could proactively assist industries and governments in Nigeria to improve food safety measures and enhance antimicrobial stewardship to curb the spread of critical antimicrobial-resistant pathogens.

## Data availability statement

The original contributions presented in the study are included in the article/**Supplementary Material**. Further inquiries can be directed to the corresponding author.

## Author contributions

EI and AB, conceptualization. EI, AB, II, AGO, and TE, formal analysis. EI and AIO, funding acquisition. EI, AB, II, AGO, TE, and AIO, investigation. EI, AB, II, and AGO, methodology. EI, AB, II, and AIO, project administration. EI and AIO, resources. EI and AIO, supervision. EI, AB, and II, roles/writing - original draft. EI, AB, II, AGO, TE, and AIO, writing - review and editing. All authors have approved the final version of the manuscript for submission. All authors agree to be accountable for all aspects of the work in ensuring that questions related to the accuracy or integrity of any part of the work are appropriately investigated and resolved.
