# Supplementary material for: Prevalence, multiple antibiotic resistance and virulence profile of methicillin-resistant Staphylococcus aureus (MRSA) in retail poultry meat from Edo, Nigeria
Source: Front Cell Infect Microbiol. 2023 Mar 2;13:1122059. doi: 10.3389/fcimb.2023.1122059 (PMC10017849; doi:10.3389/fcimb.2023.1122059)
Supplement: Supplementary file 1 [file DataSheet_1.docx]

Supplementary Material

Prevalence, Multiple Antibiotic Resistance and Virulence Profile of Methicillin-Resistant *Staphylococcus aureus* (MRSA) in Retail Poultry Meat from Edo, Nigeria

**Etinosa O. Igbinosa^1,2^*, Abeni Beshiru^1,2,3^, Isoken H. Igbinosa^1,4^, Abraham G. Ogofure^1^, Temitope C. Ekundayo^5,6^, Anthony I. Okoh^5,7^**

^1^Applied Microbial Processes & Environmental Health Research Group, Faculty of Life Sciences, University of Benin, Benin City Nigeria

^2^Stellenosch Institute for Advanced Study (STIAS), Wallenberg Research Centre at Stellenbosch University, Stellenbosch 7600, South Africa

^3^Department of Microbiology, College of Natural and Applied Sciences, Western Delta University, Oghara, Nigeria

^4^Department of Environmental Management and Toxicology, Faculty of Life Sciences, University of Benin, Benin City, Nigeria

^5^SAMRC Microbial Water Quality Monitoring Centre, University of Fort Hare, Alice 5700, Eastern Cape Province, South Africa

^6^Department of Microbiology, University of Medical Sciences, Ondo City, Ondo State, Nigeria

^7^Department of Environmental Health Sciences, College of Health Sciences, University of Sharjah, Sharjah, United Arab Emirates

*** Correspondence:**Etinosa O. Igbinosa
Etinosa.Igbinosa@uniben.edu

**Supplementary Table 1**. Primers used in this study

|  | **Target genes** | **Primer sequences (5' →3')** | **Size (bp)** | **References** |
| --- | --- | --- | --- | --- |
| *Staphylococcus aureus* | *nuc* | F:GCGATTGATGGTGATACGGTT  R:AGCCAAGCCTTGACGAACTA AAGC | 270 | Brakstad *et al*. (1992) |
| Coagulase | *coa* | F:CGAGACCAAGATTCAACAAG  R:AAAGAAAACCACTCACATCA | 730 | Aslantas *et al*. (2007) |
| Staphylococci protein A | *spa* | F:CAAGCACCAAAAGAGGAA  R:CACCAGGTTTAACGACAT | 320 | Frenay *et al*. (1996) |
| Panton valentine leucocidin | *Pvl* | F:ATCATTAGGTAAAATGTCTGGACATGATCCA R:GCATCAAGTGTATTGGATAGCAAAAGC | 433 | McClure *et al*. (2006) |
| Haemolysins | *Hla* | F:CTGATTACTATCCAAGAAATTCGATTG  R:CTTTCCAGCCTACTTTTTTATCAGT | 209 | Jarraud *et al*. (2002) |
|  | *Hlb* | F:GTGCACTTACTGACAATAGTGC  R:GTTGATGAGTAGCTACCTTCAGT | 309 | Jarraud *et al*. (2002) |
| Enterotoxins | *sea* | F:GCAGGGAACAGCTTTAGGC  R:GTTCTGTAGAAGTATGAAACACG | 521 | Monday *et al*. (1999) |
|  | *see* | F:TACCAATTAACTTGTGGATAGAC  R:CTCTTTGCACCTTACCGCA | 171 | Monday *et al*. (1999) |
|  | *Seb* | F:GGACACTAAGTTAGGGAATTATGA  R:GCTCAGTTACACCACCATAC | 200 | Mohammed *et al*., 2016 |
|  | *Sed* | F:GTGGTGAAATAGATAGGACTGC  R:ATATGAAGGTGCTCTGTGG | 385 | Pereira *et al*. (2009) |
|  | *Sec* | F:GGTATGATATGATGCCTGCAC  R:GGTGGACTTCTATCTTCACACT | 111 | Mohammed *et al*. (2016) |
|  | *seg* | F:TGTATGGTGGTGTAACTGAGCA  R:TGGTGCAGGCATCATGTCATA | 272 | Mohammed *et al*. (2016) |
|  | *seh* | F:CAACTGCTGATTTAGCTCAG  R:GTCGAATGAGTAATCTCTAGG | 359 | Pereira *et al*. (2009) |
|  | *sej* | F:CATCAGAACTGTTGTTCCGCTAG  R:CTGAATTTTACCATCAAAGGTAC | 192 | Jung *et al*. (2015) |
|  | *sei* | F:CTCAAGGTGATATTGGTGTAGG  R:AAAAAACTTACAGGCAGTCCATCTC | 577 | Jung *et al*. (2015) |
|  | *sek* | F:TAGGTGTCTCTAATAATGCCA  R:TAGATATTCGTTAGTAGCTG | 293 | Omoe *et al*. (2005) |
|  | *Sem* | F:ATGCTGTAGATGTATATGGTCTAAG  R:CGTCCTTATAAGATATTTCTACATC | 473 | Fueyo *et al*. (2005) |
|  | *sel* | F:AATATATAACTAGTGATCTAAAGGG  R:TATGGAATACTACACACCCCTTATA | 359 | Fueyo, *et al*. (2005) |
|  | *Sen* | F:ATGAGATTGTTCTACATAGCTGCAAT  R:AACTCTGCTCCCACTGAAC | 680 | Jarraud *et al*. (2002) |
|  | *seo* | F:TGTAGTGTAAACAATGCATATGCAAATG  R:TTATGTAAATAAATAAACATCAATATGATGTC | 722 | Fueyo *et al*. (2005) |
|  | *ser* | F:AAACCAGATCCAAGGCCTGGAG  R:TCACATTTGTAGTCAGGTGAACTT | 700 | Fueyo *et al*. (2005) |
|  | *seq* | F:AAGAGGTAACTGCTCAAG  R:TTATTCAGTCTTCTCATATG | 285 | Yarwood *et al*. (2002) |
|  | *sep* | F:TTAGACAAACCTATTATCATAATGG  R:TATTATCATGTAACGTTACACCGCC | 272 | Fueyo *et al*. (2005) |
|  | *Seu* | F:TAAAATAAATGGCTCTAAAATTGATGG  R:ATCCGCTGAAAAATAGCATTGAT | 141 | Letertre *et al*. (2003) |
| Toxic shock syndrome toxin 1 | *tsst*-1 | F:GCTTGCGACAACTGCTACAG  R:TGGATCCGTCATTCATTGTTAT | 559 | Monday *et al*. (1999) |
| Exfoliative toxin B precursor | *Etb* | F:ACAAGCAAAAGAATACAGCG  R:GTTTTTGGCTGCTTCTCTTG | 226 | Jackson *et al*. (1986) |
| Exfoliative toxin A precursor | *Eta* | F:GCAGGTGTTGATTTAGCATT  R:AGATGTCCCTATTTTTGCTG | 93 | Lee *et al*. (1987) |
| Intercellular adhesion protein D | *ica*D | F:ATGGTCAAGCCCAGACAGAG  R:CGTGTTTTCAACATTTAATGCAA | 198 | Arciola *et al*. (2001) |
| Intercellular adhesion protein C | *ica*C | F:TAACTTTAGGCGCATATGTTTT  R:TTCCAGTTAGGCTGGTATTG | 400 | Arciola *et al*. (2005) |
| Intercellular adhesion protein B | *ica*B | F:CTGATCAAGAATTTAAATCACAAA  R:AAAGTCCCATAAGCCTGTTT | 302 | Arciola *et al*. (2005) |
| Intercellular adhesion protein A | *ica*A | F:ACAGTCGCTACGAAAAGAAA  R:GGAAATGCCATAATGACAAC | 103 | Arciola *et al*. (2005) |
| Methicillin resistance | *mec*A | F:AAAATCGATGGTAAAGGTTGGC  R:AGTTCTGCAGTACCGGATTTGC | 532 | Strommenger *et al*. (2003) |
| Beta-lactamase | *Bla*Z | F:ACTTCAACACCTGCTGCTTTC  R:TAGGTTCAGATTGGCCCTTAG | 240 | Martineau *et al*. (2000) |
| Tetracyclines | *tet*K | F:TTAGGTGAAGGGTTAGGTCC  R:GCAAACTCATTCCAGAAGCA | 718 | Aarestrup *et al*. (2000) |
|  | *tet*L | F:TCGTTAGCGTGCTGTCATTC  R:GTATCCCACCAATGTAGCCG | 267 | Ng *et al*. (2001) |
|  | *tet*M | F:GTGGACAAAGGTACAACGAG  R:CGGTAAAGT TCG TCACACAC | 406 | Ng *et al*. (2001) |
|  | *tet*O | F:AACTTAGGCATTCTGGCTCAC  R:TCCCACTGT TCCATATCGTCA | 515 | Ng *et al*. (2001) |
| Erythromycins | *erm*A | F:TATCTTATCGTTGAGAAGGGATT  R:CTACACTTGGCTTAGGATGAAA | 139 | Martineau *et al*. (2000) |
|  | *erm*B | F:CTATCTGATTGTTGAAGAAGGATT  R:GTTTACTCTTGGTTTAGGATGAAA | 142 | Martineau *et al*. (2000) |
|  | *erm*C | F:CTTGTTGATCACGATAATTTCC  R:ATCTTTTAGCAAACCCGTATTC | 190 | Martineau *et al*. (2000) |
| Aminoglycosides | *aac(6´)-Ie-aph(2´´)-Ia* | F:CCAAGAGCAATAAGGGCATACC  R:CACACTATCATAACCATCACCG | 347 | Schmitz *et al*. (1999) |
|  | *ant(4´)-Ia* | F:CTGCTAAATCGGTAGAAGC  R:CAGACCAATCAACATGGCACC | 172 | Schmitz *et al*. (1999) |
|  | *aph(3´)-IIIa* | F:CTGATCGAAAAATACCGCTGC  R:TCATACTCTTCCGAGCAAAGG | 268 | Schmitz *et al*. (1999) |
| Chloramphenicol | *cat::p*C194 | F:CAATCCAAGGAATCATTGAAATCGG  R:AAAGCCAGTCATTAGGCCTATCTG | 472 | Argudín *et al*. (2011) |
|  | *cat::p*C221 | F:TGGAAGTTGTAAATAAAAATAAAGTG  R:CAATCCAAGGAATCATTGAAATCGG | 269 | Argudín *et al*. (2011) |
|  | *cat::p*C223 | F:AGGATATGAACTGTATCCTGCTTTG  R:AATAATGAAACATGGTAACCATCAC | 464 | Argudín *et al*. (2011) |
| Trimethoprim | *dfr*D | F:CCCTGCTATTAAAGCACC  R:CATGACCAGATAACTC | 606 | Dale *et al*. (1995) |
|  | *dfr*K | F:CAAGAGATAAGGGGTTCAGC  R:ACAGATACTTCGTTCCACTC | 229 | Argudín *et al*. (2011) |
|  | *dfr*G | F:TGCTGCGATGGATAAGAA  R:TGGGCAAATACCTCATTCC | 405 | Argudín *et al*. (2011) |
| **SCCmec** |  |  |  |  |
| Type I | *ORF E008* | F:GCTTTAAAGAGTGTCGTTACAGG  R:GTTCTCTCATAGTATGACGTCC | 613 | Zhang *et al*. (2005) |
| Type II | *kdpE* | F:GATTACTTCAGAACCAGGTCAT  R:TAAACTGTGTCACACGATCCAT | 287 | Kondo *et al*. (2007) |
| Type III | *J1 III* | F:CATTTGTGAAACACAGTACG  R:GTTATTGAGACTCCTAAAGC | 243 | Milheirico *et al*. (2007) |
| Type IVa | *ORF CQ002* | F:GCCTTATTCGAAGAAACCG  R:CTACTCTTCTGAAAAGCGTCG | 776 | Zhang *et al*. (2005) |
| Type IVb | *J1* Ivb | F:AGTACATTTTATCTTTGCGTA  R:AGTCATCTTCAATATGGAGAAAGTA | 1000 | Okuma *et al*. (2002) |
| Type IVc | *Ivc* | F:TCTATTCAATCGTTCTCGTATT  R:TCGTTGTCATTTAATTCTGAACT | 677 | Ma *et al*. (2005) |
| Type IVd | *CD002* | F:AATTCACCCGTACCTGAGAA  R:AGAATGTGGTTATAAGATAGCTA | 1242 | Kondo *et al*. (2007) |
| Type IVh | *J1* | F:TTCCTCGTTTTTTCTGAACG  R:CAAACACTGATATTGTGTCG | 663 | Milheirico *et al*. (2007) |
| Type V | *ORF V011* | F:GAACATTGTTACTTAAATGAGCG  R:TGAAAGTTGTACCCTTGACACC | 325 | Zhang *et al*. (2005) |

**Supplementary Table 2**: Antibiotic susceptibility profile of the isolates

|  |  | ***Staphylococcus aureus* (*n*=110)** | | |
| --- | --- | --- | --- | --- |
| **Antimicrobial class** | **Antibiotics** | **Resistance** | **Intermediate** | **Sensitive** |
| Penicillins | Penicillin G | 110(100) | NA | 0(0) |
| Glycopeptides | Vancomycin | 0(0) | 13(11.8) | 97(88.2) |
| Cephems | Ceftaroline | 43(39.1) | 13(11.8) | 54(49.1) |
| Lipopeptides | Daptomycin | 0(0) | 0(0) | 110(100) |
| Aminoglycosides | Gentamicin | 37(33.6) | 22(20) | 51(46.4) |
|  | Amikacin | 28(25.5) | 18(16.4) | 64(58.2) |
|  | Kanamycin | 33(30) | 21(19.1) | 56(50.9) |
| Macrolides | Azithromycin | 44(40) | 18(16.4) | 48(43.6) |
|  | Clarithromycin | 53(48.2) | 29(26.4) | 28(25.5) |
|  | Erythromycin | 49(44.6) | 26(23.6) | 35(31.8) |
| Lipoglycopeptides | Oritavancin | 0(0) | 0(0) | 110(100) |
|  | Teicoplanin | 0(0) | 0(0) | 110(100) |
| Tetracyclines | Doxycycline | 58(52.7) | 22(20) | 30(27.3) |
|  | Minocycline | 53(48.2) | 36(32.7) | 21(19.1) |
|  | Tetracycline | 64(58.2) | 32(29.1) | 14(12.7) |
| Fluoroquinolones | Ciprofloxacin | 71(64.6) | 16(14.6) | 23(20.1) |
|  | Levofloxacin | 84(76.4) | 12(10.9) | 14(12.7) |
|  | Moxifloxacin | 88(80) | 10(9.1) | 12(10.9) |
| Nitrofurantoins | Nitrofurantoin | 7(6.4) | 3(2.7) | 100(90.9) |
| Lincosamides | Clindamycin | 62(56.4) | 19(17.3) | 29(26.4) |
| Folate pathway inhibitors | Trimethoprim-  sulfamethoxazole | 39(35.5) | 13(11.8) | 58(52.7) |
|  | Sulfonamides | 53(48.2) | 13(11.8) | 44(40) |
|  | Trimethoprim | 71(64.6) | 20(18.2) | 19(17.3) |
| Phenicols | Chloramphenicol | 21(19.1) | 11(10) | 78(70.9) |
| Oxazolidinones | Linezolid | 15(13.6) | 12(10.9) | 83(75.5) |
|  | Tedizolid | 0(0) | 21(19.1) | 89(80.9) |
| Ansamycins | Rifampin | 103(93.6) | 7(6.4) | 0(0) |

**References**

Aarestrup, F.M., Agersu, Y., Ahrens, P., Jurgensen, J.C., Madsen, M., Jensen, L.B. (2000). Antimicrobial susceptibility and presence of resistance genes in staphylococci from poultry. Vet. Microbiol. 74: 353-364.

Arciola, C.R., Gamberini, S., Campoccia, D., Visai, L., Speziale, P., Baldassarri, L. (2005). A multiplex PCR method for the detection of all five individual genes of *ica* locus in *Staphylococcus epidermidis*. A survey on 400 clinical isolates from prosthesis-associated infections. J. Biomed. Mater. Res. A 75:408–413

Argudín, M.A., Tenhagen, B.A., Fetsch, A., Sachsenröder, J., Käsbohrer, A., Schroeter, A., *et al*. (2011). Virulence and resistance determinants of German *Staphylococcus aureus* ST398 isolates from nonhuman sources. Appl. Environ. Microbiol. 77:3052-3060.

Aslantas, O., Demir, C., Turutoglu, H., Cantekin, Z., Ergun, Y., Dogruer, G. (2007). Coagulase gene polymorphism of *Staphylococcus aureus* isolated from subclinical mastitis. Turkey J. Vet. Animal Sci. 31:253-257.

Brakstad, O.G., Aasbakk, K., Maeland, J.A. (1992). Detection of *Staphylococcus aureus* by polymerase chain reaction amplification of the nuc gene. J. Clin. Microbiol. 30:1654-1660.

Dale, G.E., Langen, H., Page, M.G., Then, R.L., Stüber, D. (1995). Cloning and characterization of a novel, plasmid-encoded trimethoprim-resistant dihydrofolate reductase from *Staphylococcus haemolyticus* MUR313. Antimicrob. Agents Chemother. 39:1920–1924.

Frenay, H.M., Bunschote, A.E., Schouls, L.M., van Leeuwen, W.J., Vandenbroucke-Grauls, C.M., Verhoef, J. *et al*. (1996). Molecular typing of methicillin-resistant *Staphylococcus aureus* on the basis of protein A gene polymorphism. European J. Clin. Microbiol. Infect. Dis. 15: 60-64.

Fueyo, J.M., Mendoza, M.C., Martin, M.C. (2005). Enterotoxins and toxic shock syndrome toxin in *Staphylococcus aureus* recovered from human nasal carriers and manually handled foods: Epidemiological and genetic findings. Microbes Infect. 7: 187-194.

Jackson, M.P., landolo, J.J. (1986). Sequence of the exfoliative toxin B gene of *Staphylococcus aureus*. J. Bacteriol. 167:726-728.

Jarraud, S., Mougel, C., Thioulouse, J., Lina, G., Meugnier, H., Forey, F., *et al*. (2002). Relationships between *Staphylococcus aureus* genetic background, virulence factors, *agr* groups (alleles), and human disease. Infect. Immun. 70:631–641.

Jung, S.B., Lee, Y.J., Lee, N., Kim, H.W., Oh, M., Paik, H. (2015). Virulence factors of *Staphylococcus aureus* isolated from Korean pork bulgogi: Enterotoxin production and antimicrobial resistance. Korean J. Food Sci. An. 35(4): 502-506.

Kondo, Y., Ito, T., Ma, X.X., Watanabe, S., Kreiswirth, B.N., Etienne, J., *et al*. (2007). Combination of multiplex PCRs for staphylococcal cassette chromosome *mec* type assignment: rapid identification system for *mec*, *ccr*, and major differences in junkyard regions. Antimicrob. Agents Chemother. 51: 264–274.

Lee, C.Y. Schmidt, J.J., Johnson-Winegar, A.D., Spero, L., Iandolo J.J. (1987). Sequence determination and comparison of the exfoliative toxin A and toxin B genes from *Staphylococcus aureus* J. Bacteriol. 169: 3904-3909.

Letertre, C., Perelle, S., Dilasser, F., Fach, P. (2003). Identification of a new putative enterotoxin SEU encoded by the *egc* cluster of *Staphylococcus aureus*. J. Appl. Microbiol. 95: 38-43.

Ma, X.X., Galiana, A., Pedreira, W., Mowszowicz, M., Christophersen, I., Machiavello, S., *et al*. (2005). Community-acquired methicillin-resistant *Staphylococcus aureus*, Uruguay. Emerg. Infect. Dis. 11: 973–976.

Martineau, F., Picard, F.J., Lansac, N., Menard, C., Roy, P.H., Ouellette, M., Bergeron, M.G. (2000). Correlation between the resistance genotype determined by multiplex PCR assays and the antibiotic susceptibility patterns of *Staphylococcus aureus* and *Staphylococcus epidermidis*. Antimicrob Agents Chemother. 44:231–238.

McClure, J.A., Conly, J.M., Lau, V., Elsayed, S., Louie, T., Hutchins, W., *et al*. (2006). Novel multiplex PCR assay for detection of the staphylococcal virulence marker panton-valentine leukocidin genes and simultaneous discrimination of methicillin-susceptible from -resistant staphylococci. J. Clin. Microbiol. 44:1141-1144.

Milheirico, C., Oliveira, D.C., de Lencastre, H. (2007b). Update to the multiplex PCR strategy for assignment of *mec* element types in *Staphylococcus aureus*. Antimicrob Agents Chemother. 51: 3374–3377.

Mohammed, E.Y., Abdel-Rhman, S.H., Barwa, R., El-Sokkary, M.A. (2016). Studies on enterotoxins and antimicrobial resistance in *Staphylococcus aureus* isolated from various sources. Adv Microbiol. 6: 263-275.

Monday, S.R., Bohach, G.A. (1999). Use of multiplex PCR to detect classical and newly described pyrogenic toxin genes in staphylococcal isolates. J. Clin. Microbiol. 37: 3411-3414.

Ng, L.K., Martin, I., Alfa, M., Mulvey, M. (2001). Multiplex PCR for the detection of tetracycline resistant genes. Mol. Cell Probes 15:209–215.

Okuma, K., Iwakawa, K., Turnidge, J.D., Grubb, W.B., Bell, J.M., O’Brien, F.G., *et al*. (2002). Dissemination of new methicillin-resistant *Staphylococcus aureus* clones in the community. J. Clin. Microbiol. 40:4289–4294.

Omoe, K., Dong-Liang, H., Takahashi-Omoe, H., Nakane, A., Shinagawa, K. (2005). Comprehensive analysis of classical and newly described staphylococcal super antigenic toxin genes in *Staphylococcus aureus* isolates. FEMS Microbiol. Lett. 246: 191-198.

Pereira, V., Lopes, C., Castro, A., Silva, J., Gibbs, P., Teixeira, P. (2009). Characterization for enterotoxin production, virulence factors, and antibiotic susceptibility of *Staphylococcus aureus* isolates from various foods in Portugal. Food Microbiol. 26: 278-282.

Schmitz, F.J., Fluit, A.C., Gondolf, M., Beyrau, R., Lindenlauf, E., Verhoef, J., *et al*. (1999). The prevalence of aminoglycoside resistance and corresponding resistance genes in clinical isolates of staphylococci from 19 European hospitals. J. Antimicrob Chemother. 43:253–259.

Strommenger, B., Kettlitz, C., Werner, G., Witte, W. (2003). Multiplex PCR assay for simultaneous detection of nine clinically relevant antibiotic resistance genes in *Staphylococcus aureus*. J. Clin. Microbiol. 41: 4089-4094.

Yarwood, J.M., McCormick, J.K., Paustian, M.L., Orwin, P.M., Kapur, V., Schlievert, P.M. (2002). Characterization and expression analysis of *Staphylococcus aureus* pathogenicity island 3 - Implications for the evolution of staphylococcal pathogenicity islands. J. Biol. Chem. 277:13138-13147.

Zhang, K., McClure, J.A., Elsayed, S., Louie, T., Conly, J.M. (2005). Novel multiplex PCR assay for characterization and concomitant subtyping of *Staphylococcus* cassette chromosome *mec* types I to V in methicillin-resistant *Staphylococcus aureus*. J. Clin. Microbiol. 43:5026–5033.
